# Cuba in Mexico: first record of *Phyllops falcatus* (Gray, 1839) (Chiroptera, Phyllostomidae) for Mexico and other new records of bats from Cozumel, Quintana Roo

**DOI:** 10.3897/zookeys.973.53185

**Published:** 2020-10-05

**Authors:** Noel Anselmo Rivas-Camo, Paulina Abigail Sabido-Villanueva, Carlos Ricardo Peralta-Muñoz, Rodrigo A. Medellin

**Affiliations:** 1 Centro de Conservación y Educación Ambiental de la Fundación de Parques y Museos de Cozumel, Quintana Roo, Av. Pedro Joaquin Coldwell entre primera sur y Juarez, No. 70, Colonia Centro, C.P. 77600, Cozumel, Quintana Roo, México Centro de Conservación y Educación Ambiental de la Fundación de Parques y Museos de Cozumel Cozumel Mexico; 2 Instituto de Ecología, UNAM, Ap. Postal 70-275, Coyoacán, 04510 Ciudad de México, México Instituto de Ecología México Mexico

**Keywords:** Caribbean islands, Cuban archipelago, dispersal, hurricanes, range extension, Yucatan Peninsula

## Abstract

The first record of *Phyllops falcatus* (Gray, 1839) in Mexico is documented from the island of Cozumel, Quintana Roo. This species is present in the Antilles, distributed in all the Cuban archipelago, Cayman Islands, and Hispaniola. It is likely that a hurricane moved these bats from Cuba to Cozumel. The Cozumel record extends the distribution more than 200 km west. Two new records from Cozumel of the bats *Lasiurus ega* and *Molossus alvarezi* are also provided.

## Introduction

Cozumel is an island in the Mexican Caribbean with an area of about 647 km^2^ and about 20 km off the coast of the state of Quintana Roo (Orellana et al. 2007). It is the Mexican island with not only the greatest species richness but also the highest endemism of any island of the country (ECOSUR 2018). However, there are few works addressing the bat diversity of Cozumel. Koopman (1959), based on the then known 19 species of mammals of Cozumel, outlined the limits of the West Indies and discarded Cozumel as an island that does not belong to the Antilles subregion, as all the mammals known in Cozumel at the time had affinities with the Yucatan Peninsula or were undetermined. Jones and Lawlor (1965) recorded eight species of bats and their conclusions were similar, that Cozumel is a continental island with affinities to Yucatan, and not to the Antilles, because of the absence of endemic Antillean species. CONABIO (2008) presented a list of 22 species of bats for the island, although several of them are undoubtedly erroneous, like *Glossophaga leachii* Gray, 1844 and *Rhogeessa parvula* H. Allen, 1866, which are only found on the Pacific tropical dry forest (Medellin et al. 2008) and *Artibeus intermedius* J. A. Allen, 1897 that has been included as a synonym of *A. lituratus* (Olfers, 1818) (Simmons 2005). Similarly, Rincón-Sandoval (2013) reported a list of 22 species from Cozumel, including three new records that we consider dubious for the island. *Molossus sinaloae* J. A. Allen, 1906 was recorded from echolocation recordings, but it is well known that the family Molossidae features great diversity in the design of their echolocation calls, and that their calls are similar within the genus (Jung et al. 2014). However, this report of *M. sinaloae* may have instead been *M. alvarezi*González-Ruiz et al. (2011). Likewise, the echolocation call of *Pteronotus davyi* Gray, 1838 can be confused with that of *P. gymnonotus* (J. A. Wagner, 1843) and also possibly with *P. personatus* (J. A. Wagner, 1843) (Ibañez et al. 2000). Orozco-Lugo (pers. comm. to N. Rivas 2000, 2006) by means of mist nets and ultrasound detectors reported the possible presence of *Lasiurus ega* (Gervais, 1856), *Diphylla ecaudata* Spix, 1823, and *Mormoops megalophylla* (Peters, 1864) on Cozumel. However, the calls of *Lasiurus intermedius* H. Allen, 1862 can also be confused with those of other congeneric species. Given that there are no specimens that backup the correct identification of these species, we consider their presence unconfirmed in Cozumel.

In previous lists, *Corynorhinus mexicanus* G. M. Allen, 1916 has been reported from Cozumel, but Koopman (1959), Jones and Lawlor (1965), and other authors have clarified that *Corynorhinus* sp. is not present in Cozumel or even the Yucatan Peninsula. Likewise, *Lasiurus borealis* (Müller, 1776) only exists in northeastern Mexico (Medellin et al. 2008).

Previous to this study, the only species of the genus *Molossus* known from Cozumel was *M. rufus* É. Geoffroy 1805. González-Ruiz et al. (2011) described a new species of the genus *Molossus* for the populations from the Yucatan Peninsula formerly ascribed to *M. sinaloae*. They described *M. alvarezi* on the basis of morphometric characters that clearly distinguished it as a species, endemic to Yucatan.

The genus *Phyllops* (Chiroptera, Phyllostomidae) is endemic to the Antilles (Da Cunha and Mancina 2008). This genus has one living species, *Phyllops falcatus* (Gray, 1839), with two subspecies, *P. falcatus falcatus* that inhabits Cuba, Grand Cayman, and Cayman Brac, and *P. falcatus haitiensis* in Hispaniola. The genus also has an extinct species, *P. vetus*, from Cuba and Isla de la Juventud (Silva 1979; Suárez and Díaz-Franco 2003). This species is assessed as Least Concern by the IUCN (Solari et al. 2019). *Phyllops* can be distinguished from other Caribbean species by the presence of three upper molars, well-developed post-orbital processes, a small gap between the incisor foramina and the incisor root, and a broad noseleaf that is broader in its midsection than at its base. The skull is tall and round, without a developed sagittal crest, and with a well-developed metaconid in m1 (Da Cunha and Mancina 2008). With the objective to contribute to the understanding and knowledge of the bat fauna of the island, we provide an updated list of bats of Cozumel and report for the first time the presence of three species, including one that is new for Mexico (Table 1).

## Material and methods

### Area of study

Cozumel is the largest island of Mexican Caribbean and is comprised of limestone. The soil is very permeable, which causes the water to rapidly filter (Téllez-Valdez et al. 1989), and due to this, the flow of water is primarily subterranean (Alcocer and Escobar 1996). The coastal ecosystems of the Yucatan Peninsula have three well-defined seasons: the dry season from March to May, the hot rainy season from June to October, and cold rainy season from November to February, which is driven by cold fronts (Herrera-Silveira et al. 2010). The annual total precipitation varies between 800 and 1500 mm. The driest months are March and April; September is the month with the most of rain due to the passage of hurricanes (Orellana et al. 2007). Cozumel has the Am(f) climate subtype, which is characterized by a warm, humid summer with rain. The annual average temperature is between 26 and 27 °C (Orellana et al. 2007).

The flora of the island is only 40% of that reported from the state of Quintana Roo (Téllez-Valdez et al. 1989). Dominant vegetation in Cozumel is middle subdeciduous forest. The island also has low subdeciduous forest, magrove forest, and wetlands with reeds, palms, etc., and halophyte vegetation or coastal dune (Téllez-Valdez et al. 1989). The island also has several endemic vertebrates such as pygmy Cozumel raccoon (*Procyon pygmaeus* Merriam, 1901), Cozumel harvest mouse (*Reithrodontomys spectabilis* Jones & Lawlor, 1965), Cozumel emerald (*Chlorostilbon forficatus* Ridgway, 1885), Cozumel vireo (*Vireo bairdi* Ridgway, 1885) among other species (Jones and Lawlor 1965; McFadden et al. 2006; Aves de Cozumel 2020).

### Field surveys

As part of an ongoing biological inventorying of mammals and birds, field surveys have been conducted for the past 3.5 years. We started in February 2017 using 12-m long mist nets. Sampling sites were Parque Ecológico Estatal Laguna Colombia, El Cedral, and the San Gervasio Archeological Site. Parque Ecológico Estatal Laguna Colombia is located in southwestern Cozumel. It is covered by coastal dune vegetation, subdeciduous low forest, and mangroves with a brackish lagoon. El Cedral is a small human community that is also located to the southwest, activities of agriculture and livestock as well as human habitation occur in the settlement. The San Gervasio Archeological Site is 7 km east of the city of San Miguel de Cozumel. Both El Cedral and San Gervasio sites are covered by middle subdeciduous forest.

On April 5, 10, and 25, 2019, we captured bats in the San Gervasio Archaeological Site. Dominant arboreal species included: *Manilkara zapota*, *Bursera simaruba*, *Cedrela odorata* L. (1759), *Metopium brownei*, *Ceiba aesculifolia*, *Lysiloma latisiliquum*, and *Sideroxylon foetidissimum* (Téllez-Valdez et al. 1989).

We set two or three 12 m long mist nets, fixed with poles and cords, in the vegetation on April 25, 2019 and on March 2, 2020. On each capturing night, we opened the nets at 19:00 hr and closed them at 00:00 hr. The species were identified using the guide by Medellin et al. (2008). An additional bat record came from a local inhabitant who had found a dead bat on October 6, 2017. We visited the location which was at km 4.5 of the road to San Gervasio and collected the specimen. All bats were handled according to the guidelines for the use of wild mammals in research (Sikes et al. 2016).

To compile the list of bat species present in Cozumel, we examined all the literature containing reports of bats from the island. In addition, we added our own information, the results of the ongoing inventory, and talked to other researchers working on the island. Bat nomenclature and taxonomy follows Ceballos et al. (in press).

## Results

**Figure 1.**
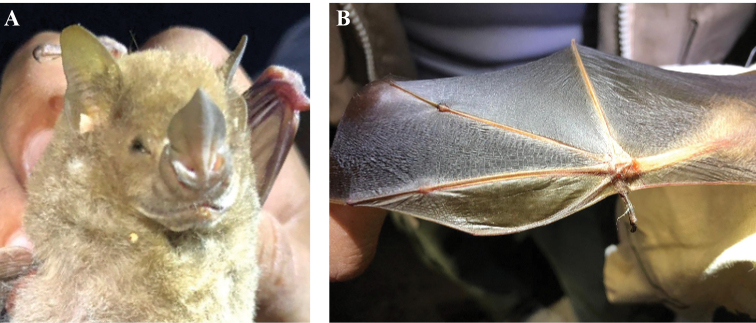
*Phyllops falcatus* from an area adjacent to the San Gervasio Archeological Site, Cozumel island, Mexico **A** lactating female showing the noseleaf that is broader in its midsection than its base **B** recurved second finger and translucent dactylopatagium minus. Photograph by P. Sabido (**A**) and N. Rivas (**B**).

During our first field survey on April 5, 2019, we captured a bat that did not match any species known from México (Ceballos and Oliva 2005; Medellin et al. 2008). This bat was a lactating female. It had two white spots on the shoulders, the central part of the noseleaf was wider than its base, the tragus was yellowish, and the forearm length was 49 mm (Fig. 1). The second metacarpal of the index finger was curved and the first dactylopatagium was wide and translucent. This bat had three upper molars. The specimen was photographed, weighed, morphometric measurements were obtained using manual calipers and then it was released.

We identified this bat using a field key including the four short-faced stenodermatine genera found on the Antilles, and it was identified as *Phyllops falcatus*. On April 10, we caught three adult specimens of *P. falcatus*. Their forearm lengths were 45, 42, and 44 mm and body masses were 17, 17.8, 20.3 g, for one male with scrotal testes, one non-reproductive male, and one non-reproductive female, respectively. The length of the thumb was 10 mm for all three specimens. We collected one of these males, which was deposited in Mexico’s Colección Nacional de Mamíferos at the Institute of Biology (UNAM). Unfortunately the skull was lost. On April 25 an additional non-reproductive female of *Phyllops falcatus* was caught; it had a forearm of 47 mm and body mass of 14.5 g. All five individuals of *P. falcatus* were caught between 20:38 and 21:51 hrs.

On February 22, 2019, we captured an adult male *Lasiurus ega*, with a forearm of 43.5 mm and body mass of 19 g. This is the first specimen of this species to be captured on the island. Finally, on March 2, 2020, we captured an adult male *Molossus alvarezi*, which is clearly distinguishable from the other *Molossus* known from the island by its smaller size. This species was considered endemic to the north and east parts of the Yucatan Peninsula, and was not known from Cozumel. A subsequent study by Loureiro et al. (2019) extended its distribution to Central America and French Guiana in northern South America. The specimen reported on Oct 6, 2017 was determined to be a male *Lasiurus frantzii* Peters, 1870, the first individual of this species to be found on Cozumel in 100 years since Gaumer collected one individual on the island (Koopman 1959).

The following species were recorded from February 2017 to August 2019 from the Punta Sur area: *Rhogeessa aeneus* Goodwin, 1958, *Myotis pilosatibialis* LaVal, 1973, *Artibeus jamaicensis* Leach, 1821, and *Natalus mexicanus* Miller, 1902. From March 2019 to March 2020, the species captured at the San Gervasio site were: *Artibeus jamaicensis*, *Artibeus phaeotis* (Miller, 1902), *Artibeus lituratus*, *Centurio senex* Gray, 1842, *Glossophaga soricina* (Pallas, 1766), *Phyllops falcatus*, *Pteronotus mesoamericanus* Smith, 1972, *Molossus alvarezi*, *Eptesicus furinalis* (d’Orbigny, 1847), *Myotis pilosatibialis*, *Rhogeessa aeneus*, and *Lasiurus ega*. Lastly, in the zone of El Cedral, from March 2019 to December 2019, we recorded these species: *Artibeus jamaicensis*, *Centurio senex*, *Micronycteris microtis* Miller, 1898, *Rhogeessa aeneus*, *Myotis pilosatibialis*, *Eptesicus furinalis*, and *Pteronotus mesoamericanus*. This brings the number of bats known from Cozumel to 19 (Table 1).

**Table 1.** Updated list of the 19 bat species from Cozumel, Mexico including the first reports of each species in the island. * indicates species newly recorded from the island as per our work.

Species	First record for Cozumel	NOM-059 Status
**Family Phyllostomidae**		
*Artibeus jamaicensis*	Koopman 1959	
*Artibeus lituratus*	Jones and Lawlor 1965	
*Artibeus phaeotis*	Jones and Lawlor 1965	
*Centurio senex*	Koopman 1959	
*Glossophaga soricina*	Koopman 1959	
*Micronycteris microtis*	Jones and Lawlor 1965	
*Mimon cozumelae*	Goldman 1914	THREATENED
Phyllops falcatus*	This study	
**Family Mormoopidae**		
*Pteronotus mesoamericanus*	Engstrom et al. 1989	
**Family Natalidae**		
*Natalus mexicanus*	Jones and Lawlor 1965	
FAMILY MOLOSSIDAE		
*Eumops bonariensis*	Engstrom et al. 1989	
*Molossus alvarezi* *	This study	
*Molossus rufus*	Koopman 1959	
*Nyctinomops laticaudatus*	Koopman 1959	
**Family Vespertilionidae**		
*Eptesicus furinalis*	Rincón-Sandoval 2013	
*Lasiurus frantzii*	Koopman 1959	
*Lasiurus ega* *	This study	
*Myotis pilosatibialis*	LaVal 1973	
*Rhogeessa aeneus*	Jones et al. 1973	

## Discussion

The distance between the northeastern point of Cozumel and the western limit of the distribution of *P. falcatus* in Villa Cabo San Antonio in western Cuba is 235 km, separated by the Caribbean Sea. Furthermore, the distance between Villa Cabo San Antonio and Cancun is less than 210 km and without and islands or islets of any kind. This distance is too far for *P. falcatus* to disperse by normal flight, given its assumed flight capability. For example, a related stenodermatine, *Sturnira lilium* (E. Geoffroy, 1810), is known to move a maximum of 760 m from roost to foraging area (Mello et al. 2008). Another phyllostomid, the insectivorous trawler *Macrophyllum macrophyllum* (Schinz, 1821), can move 7 km from the roost, although in foraging bouts it may reach up to 47 km, a distance is considered extreme (Meyer et al. 2005), but less than a quarter of the distance from Cuba to Cozumel. We speculate that *P. falcatus* could have been carried by a hurricane that passed by Cuba and continued its path to the Yucatan Peninsula and Cozumel. Both Cuba and Cozumel are located in the Caribbean Hurricane Alley. From 2000 to 2016, more than 20 hurricanes passed over Cuba (Hypothetical hurricanes 2020). From 1879 to 2016, 34 hurricanes passed over the Yucatan Peninsula, including Cozumel Island (Aves de Cozumel 2020).

Three invasion routes for overwater dispersal of bats in the Caribbean have been proposed and identified as the northern, western, and southern routes (Rodriguez-Duran and Kunz 2001). However, these routes were proposed for mainland species invading Caribbean islands, and *P. falcatus* has clearly dispersed in the reverse direction, coming from the Caribbean island of Cuba and invading the mainland. Hurricanes seem to have a severe negative effect on bat and bird populations (Pedersen et al. 1996; Rodríguez-Durán and Kunz 2001), but clearly for *P. falcatus*, hurricanes have allowed this species to reach and colonize a new area. It is likely that *P. falcatus* may continue to disperse and easily reach the mainland of the Yucatan Peninsula. Based on the available data, we conclude that passive dispersal by tropical storms, not purposeful bat dispersal behavior, are responsible for insular dispersal and gene flow of bat populations living in the Lesser Antilles (Pedersen et al. 2013).

The presence of *P. falcatus* in Cozumel is not incidental, given that we have captured a total of five specimens, and that the very first specimen was a lactating female. This indicates that the species is present in the island with a breeding population. Given the geography of the Caribbean, and the presence of *P. falcatus* in Cozumel, it is possible that other species of bats, notably *Ardops*, *Stenoderma*, and *Ariteus* may also be recorded in the future. Likewise, we predict that with more surveys, *P. falcatus* is likely to be recorded in the vicinity of Cancun.

Given its recent detection in Mexico and that this may be the only population in the country, this species may warrant conservation status of at least Threatened under Mexican legislation. We are in the process of conducting genetic studies to determine the relationship of animals from Cozumel with those from Cuba. The presence of this species increases the number of known bat species in Mexico to 140.

Very little is known still about bats of Cozumel over time because there are fewer than five publications documenting them and many errors in labeling and identification of specimens. Information on the species richness of this group in Cozumel is muddled. The bad reputation that these flying mammals have with the general public makes educating the people of Cozumel about bats, the value of the island’s fauna, and the importance of bat conservation necessary.
